# Association between short stature and behavioral and emotional difficulties among children in Jordan: a cross-sectional study

**DOI:** 10.3389/fendo.2025.1630919

**Published:** 2025-08-25

**Authors:** Tamara Kufoof, Randa K. Saad, Redab Al-Ghawanmeh, Seri Sawaqed, Zaid Hamdan, Zaid Qolaghasi, Adnan Alswiti, Layan Sharkas, Osama Sharkas

**Affiliations:** ^1^ Department of Pediatrics, Faculty of Medicine, The Hashemite University, Zarqa, Jordan; ^2^ Research and Policy, Eastern Mediterranean Public Health Network (EMPHNET), Amman, Jordan; ^3^ Department of Internal Medicine, Faculty of Medicine, The Hashemite University, Zarqa, Jordan

**Keywords:** short stature, Jordan, behavioral difficulties, emotional difficulties, Strengths and Difficulties Questionnaire (SDQ)

## Abstract

**Background:**

Short stature is associated with psychological and emotional challenges, yet its impact on children’s behavioral well-being in Jordan remains underexplored. This study examines the association between short stature and behavioral and emotional difficulties in Jordanian children.

**Methods:**

A cross-sectional study was conducted at Prince Hamza Hospital, Amman (2023–2024). We recruited eighty-three children aged 4–14 years with a height percentile ≤3%. The Strengths and Difficulties Questionnaire (SDQ) assessed behavioral and emotional difficulties. Differences in SDQ scores by gender, age group, and growth hormone (GH) therapy status were analyzed.

**Results:**

The mean total difficulties score was 13.7 ± 6.1, with 30.1% of participants classified in the “high” or “very high” category. Emotional difficulties (31.3%), peer problems (31.3%), and conduct issues (34.9%) were notably prevalent. Boys exhibited significantly higher conduct problems (p < 0.001), hyperactivity (p = 0.002), and total difficulties scores (p = 0.010), while girls showed stronger prosocial behaviors (p = 0.004). No significant differences were observed between younger (3–10 years) and older (11–14 years) children. Children receiving GH therapy reported significantly higher emotional problems (p = 0.029), though other SDQ scores did not differ significantly between treated and untreated groups.

**Conclusion:**

Short stature in Jordanian children is associated with significant behavioral and emotional challenges, particularly among boys. GH therapy was not associated with improved scores in this sample. A multidisciplinary approach integrating psychological support and public health initiatives is needed to address stigma and improve well-being.

## Background

Short stature is defined as a condition where an individual’s height is below the third percentile or at least two standard deviations (SD) below the mean height for their sex and chronological age in a given population (1). This condition can either result from normal variations in growth, the most common cause, or signify underlying pathological concerns (2). Pathological short stature is often associated with health-related stress, stemming from the effects of known or unknown medical conditions on quality of life (3). Nevertheless, even in children with idiopathic short stature, the condition can impose significant psychological and emotional challenges for both the child and their parents, irrespective of the underlying cause (4, 5).

The psychosocial implications of short stature include physical challenges, such as limited accessibility or exclusion from certain physical activities, which can lead to feelings of inadequacy (6). Social dynamics, including bullying, negative stereotyping, and societal biases linking positive attributes to height, further compound the psychological burden (7, 8). Children with short stature often experience diminished self-esteem, body image concerns, and heightened vulnerability to discrimination (7, 8). Furthermore, parents may overcompensate by being overly protective (9), inadvertently fostering behavioral problems, and perpetuating a sense of helplessness in their child (10), including low self-esteem and self-perception (11). These challenges cumulatively affect the child’s overall psychological functioning and quality of life.

By definition, approximately 2.5% of the global population falls below two standard deviations from the median height-for-age, classifying them as having short stature. However, prevalence rates can vary based on genetic and environmental factors (12). In Jordan, a nationwide study conducted in 2016 reported a prevalence of 4.9% among children aged 6–17 years (13). However, there remains a significant gap in understanding the psychological effects of short stature within the Jordanian context. Insights into these effects are critical, as they impact children’s immediate quality of life and shape their long-term social and emotional development.

This study aims to address this knowledge gap by investigating the associations between short stature and psychological well-being from the perspective of patients and their parents. By evaluating psychosocial functioning, this research seeks to provide a nuanced understanding of the psychological challenges faced by children with short stature in Jordan, thereby informing interventions to improve their quality of life.

## Methods

### Study design

This is a cross-sectional study conducted at the pediatric endocrinology clinic at Prince Hamza Hospital, a tertiary care referral hospital in central Amman, Jordan. The recruitment period lasted for ten months during the years 2023 and 2024.

### Population

Any child attending the clinic for short stature assessment with a height percentile less than or equal to 3%, as per the US Centres for Disease Control and Prevention Age-Height growth charts (14), was asked to join the study.

The following formula was used to determine the sample size: n = (Z² × P × (1 - P))/E². Here, Z represents the z-score at a 95% confidence level, P is the estimated prevalence of short stature in Jordan (4.9%) (13), and E is the desired margin of error (5%). This gave us a sample size of around 74. As the study was exploratory and not powered for hypothesis testing across subgroups, we recruited a sample of 83 children aged between 4 and 14 years, which exceeded our initial estimate.

### Instrument

We used the Strengths and Difficulties Questionnaire (SDQ) to evaluate the behavioral and emotional difficulties of children with short stature. The SDQ is a widely utilized 25-item questionnaire that captures both strengths and difficulties across five domains (subscales): Emotional Symptoms, Conduct Problems, Hyperactivity/Inattention, Peer Relationship Problems, and Prosocial Behaviour (15). Each question is rated on a three-point scale (0 = not true, 1 = somewhat true, 2 = certainly true) (16).

In addition to generating subscale scores, the SDQ provides a Total Difficulties Score as well as Internalizing and Externalizing Scales. The Total Difficulties Score is a sum of scores from all scales except the prosocial scale. It provides an overall measure of behavioral and emotional difficulties. The Internalizing Scale combines Emotional Symptoms and Peer Relationship Problems, offering insights into internalizing difficulties such as anxiety and depression. The Externalizing Scale combines Conduct Problems and Hyperactivity/Inattention, providing an assessment of externalizing behaviors like aggression and hyperactivity. An optional Impact Supplement assesses the perceived distress and social impairment associated with identified difficulties.

The SDQ was chosen for its brevity, multidimensional structure, and ease of administration in clinical interviews. Its Arabic version has been used extensively across the region in research and clinical settings. While a formal validation study of the Arabic SDQ has not been conducted specifically in Jordan, it has demonstrated acceptable psychometric performance in Arab-speaking populations more broadly (17).

### Data collection

Eligible patients were identified by the treating physician based on the child’s age and diagnosis of short stature. Detailed information about the study, its purpose, procedures, and potential risks and benefits was provided to the parents and children, as appropriate.

Trained data collectors administered the SDQ individually through an interview, where questions were explained and translated into Arabic, the parents’, and participants’ native language, to ensure participants’ comprehension and accurate responses. Translation of questions was performed by bilingual and culturally competent interviewers to maintain accuracy and cultural sensitivity. Collected data was recorded, anonymized, and securely stored in an electronic tool.

We also conducted a retrospective review of the participants’ hospital medical records to collect information on the participants’ underlying diagnosis of short stature, relevant medical history, height, weight, and the heights of both parents.

### Data analysis

We summarized participant characteristics using frequencies for categorical variables and calculated means ± SD with (minimum, maximum) for continuous variables.

To calculate the mean score for each of the SDQ domains, we added the scores of the five questions within that domain. The highest stage of each domain corresponds to a score of 10. We also calculated the Total Difficulty score by combining the scores for each of the difficulties; this gives a score range from 0 to 40. Similarly, we calculated the Externalizing score, which ranges from 0 to 20, by combining the conduct and hyperactivity scales. We calculated the internalizing score, which also ranges from 0 to 20, by combining the emotional and peer problems scales. We used descriptive statistics to calculate means ± SD and medians (minimum, maximum) for each of the domains.

We used the thresholds recommended by the SDQ as cut-off points. These thresholds are based on the cut-offs above which participants are classified into the High or Vey High category of a four-band SDQ classification scheme based on a UK population survey. This band classifies 80% of the population as ‘close to average’, 10% as ‘slightly raised’, 5% as ‘high’, and 5% as ‘very high’ for all scales except prosocial, which is 80% ‘close to average’, 10% ‘slightly lowered’, 5% ‘low’ and 5% ‘very low’ (18).

We tested all scores for gender differences, age group differences, and differences based on whether the child received growth hormone (GH) replacement or not, using Independent t-test. To examine differences in the percentage of those who scored within the clinical range, we used Chi-square tests. A significance level of.05 was applied to all statistical tests.

We rounded numbers to the nearest integer unless otherwise specified. SPSS version 26 (IBM, Chicago, IL) was used for analyses.

## Results

### Patient demographics

The study included 83 patients, with 48.2% female. The mean age was 10.4 ± 2.5 years; 34 children (41.0%) were in the 3–10 year range, and 49 (59.0%) were between 11 and 14 years. The mean height percentile was 1.2 ± 0.9. The sex-adjusted mid-parental height had a mean of 163.0 ± 7.9 cm.

A total of 22 patients (26.5%) had a history of GH replacement. The most common diagnoses were familial short stature (FSS), with 12 (14.5%) patients having isolated FSS and nine other patients having FSS in combination with another diagnosis. Isolated constitutional delay in growth and puberty (CDGP) and isolated idiopathic short stature (ISS) were each present in 10 (12.0%) individuals, while isolated growth hormone deficiency (GHD) was present in nine (10.8%) individuals (**Table 1**).

**Table 1:** Baseline characteristics, N=83.

Patient characteristics	N (%) or Mean ± SD, (min, max)
Sex	
Males	43 (51.8)
Females	40 (48.2)
Age, years	10.4 ± 2.5, (4,14)
Age groups, years	
3-10	34 (41.0)
11-14	49 (59.0)
Height percentile, %	1.2 ± 0.9, (0.1,3.0)
Weight percentile, %	9.1 ± 16.0, (0.1, 97.0)
Body Mass Index percentile, %	35.3 ± 29.9, (0.0, 99.7)
Sex-adjusted mid parental height^1^, cm	163.0 ± 7.9, (146.0, 177.7)
History of GH replacement	
Yes	22 (26.5)
No	61 (73.5)
Most likely diagnosis	
FSS	12 (14.5)
FSS + IUGR	1 (1.2)
FSS + SGA	3 (3.6)
FSS + T1D	1 (1.2)
FSS + CDGP	2 (2.4)
GHD	9 (10.8)
GHD + FSS	2 (2.4)
GHD + SGA	1 (1.2)
GHD + FMF	1 (1.2)
GHD + craniosynostosis	1 (1.2)
CDGP	10 (12.0)
ISS	10 (12.0)
SGA	5 (6.0)
Celiac disease	5 (6.0)
Celiac disease + T1D	1 (1.2)
Celiac disease + Hashimoto	1 (1.2)
Celiac disease + SGA	1 (1.2)
Turner syndrome	5 (6.0)
IUGR + congenital hypothyroidism	1 (1.2)
Syndromic (Unknown)	1 (1.2)
Barter Syndrome	1 (1.2)
Silver-russel syndrome	1 (1.2)
Under investigation	8 (9.6)

CDGP, Constitutional delay in growth and puberty; FMF, Familial Mediterranean Fever; FSS, Familial short stature; GHD, Growth Hormone deficiency; ISS, Idiopathic short stature; IUGR, Intrauterine growth retardation; SGA, Small for gestational age; T1D, Type 1 Diabetes. ^1^Sex adjusted mid parental height was calculated as: For Female = ((Height of Father - 13) + Height of Mother)/2; and for Males = ((Height of Mother + 13) + Height of Father)/2(18).

### Psychological difficulties and behavioral profiles

**Table 2** presents the scores for the different SDQ domains. The mean total difficulties score was 13.7 ± 6.1, with 30.1% of children classified in the “high” or “very high” category. The mean internalizing score, comprising emotional and peer problems, was 6.4 ± 3.3, with 22.9% scoring in the “high” or “very high” category. The mean externalizing score, which includes conduct problems and hyperactivity, was 7.3 ± 4.1, with 16.9% exceeding the “high” or “very high” range cut-off.

**Table 2:** Scores of the different domains of the strengths and difficulties questionnaire in children with short stature, n=83.

Domain	Overall score		Percentile					Cut-off points^1^	% above cut-off
	Mean ± SD	Median (min, max)	10	25	50	75	90		
Emotional problems	3.5 ± 2.2	3.0 (0.0, 9.0)	1	2	3	5	7	≥ 5	31.3
Conduct problems	2.8 ± 2.1	2.0 (0.0,8.0)	0	1	2	4	6	≥ 4	34.9
Hyperactivity	4.4 ± 2.5	4.0 (0.0, 9.0)	1	3	4	6	8	≥ 8	18.1
Peer problems	2.9 ± 1.9	3.0 (0.0, 9.0)	1	2	3	4	5.6	≥ 4	31.3
Prosocial	8.4 ± 2.1	9.0 (0.0, 10.0)	5	8	9	10	10	≤ 6	14.5
Total difficulties	13.7 ± 6.1	13.0 (2.0, 30.0)	7	9	13	17	22	≥ 17	30.1
Internalizing	6.4 ± 3.3	6.0 (1.0, 15.0)	2	4	6	8	12	≥ 9	22.9
Externalizing	7.3 ± 4.1	7.0 (0.0, 17.0)	3	4	7	10	13	≥ 12	16.9
Impact	1.4 ± 3.0	0.0 (0.0, 10.0)	0	0	0	0	6.6	≥ 2	24.1

Total difficulties score: Sum of scores of all scales except the prosocial scale. The resultant score ranges from 0 to 40. Externalizing scores: Sum of the conduct and hyperactivity scales, and ranges from 0 to 20. Internalizing scores: Sum of the emotional and peer problems scales, and ranges from 0 to 20. ^1^The cut-off point above which participants are classified into the High or Vey High category of a four-band SDQ classification scheme, that is based on a UK population survey. This band classifies 80% of the population as ‘close to average’, 10% as ‘slightly raised’, 5% as ‘high’, and 5% as ‘very high’ for all scales except prosocial, which is 80% ‘close to average’, 10% ‘slightly lowered’, 5% ‘low’ and 5% ‘very low’.

Among the individual domains, emotional problems had a mean score of 3.5 ± 2.2, with 31.3% of participants classified in the “high” or “very high” category. Similarly, peer problems had a mean score of 2.9 ± 1.9, with 31.3% exceeding the cut-off. Conduct problems had a mean score of 2.8 ± 2.1, with 34.9% in the “high” or “very high” range, while hyperactivity had a mean score of 4.4 ± 2.5, with 18.1% exceeding the cut-off.

The prosocial domain showed a mean score of 8.4 ± 2.1, with 14.5% of children scoring in the “low” or “very low” category. The impact score, which reflects the perceived burden of difficulties, had a mean value of 1.4 ± 3.0, with 24.1% of participants scoring above the cut-off point.

### Gender differences

As shown in **Table 3**, males demonstrated significantly higher mean scores for conduct problems (3.7 ± 2.1 vs. 1.9 ± 1.7, p < 0.001), hyperactivity (5.2 ± 2.7 vs. 3.6 ± 1.9, p = 0.002), and total difficulties (15.3 ± 6.3 vs. 11.9 ± 5.4, p = 0.010). Additionally, externalizing scores were markedly higher in males (9.0 ± 4.3 vs. 5.4 ± 2.8, p < 0.001).

**Table 3:** Scores of the different domains of the strengths and difficulties questionnaire in children with short stature, by sex, n females= 40 and n males =43.

Domain	Female scores	Male scores	P Value	% Above cut-off^1^		P value
	Mean ± SD	Mean ± SD		Females N=40	Males N=43	
Emotional problems	3.5 ± 2.4	3.4 ± 2.1	0.866	32.5	30.2	0.824
Conduct problems	1.9 ± 1.7	3.7 ± 2.1	**< 0.001**	17.5	51.2	**0.001**
Hyperactivity	3.6 ± 1.9	5.2 ± 2.7	**0.002**	5.0	30.2	**0.003**
Peer problems	3.0 ± 1.6	2.9 ± 2.2	0.919	30.0	32.6	0.802
Prosocial	9.1 ± 1.5	7.8 ± 2.4	**0.004**	7.5	20.9	0.082
Total difficulties	11.9 ± 5.4	15.3 ± 6.3	**0.010**	20.0	39.5	0.053
Internalizing	6.5 ± 3.4	6.4 ± 3.3	0.865	25.0	20.9	0.659
Externalizing	5.4 ± 2.8	9.0 ± 4.3	**< 0.001**	2.5	30.2	**0.001**
Impact	1.2 ± 2.8	1.7 ± 3.1	0.446	20.0	27.9	0.400

^1^ For the Prosocial domain, ‘low’ and ‘very low’ categories were defined as those below the cutoff score of 6.

Values in bold indicate a significant result.

More males scored above the thresholds for conduct problems (51.2% vs. 17.5%, p = 0.001), hyperactivity (30.2% vs. 5.0%, p = 0.003), and externalizing scores (30.2% vs. 2.5%, p = 0.001). While females had significantly higher prosocial scores (9.1 ± 1.5 vs. 7.8 ± 2.4, p = 0.004), the percentage of those below the prosocial cutoff (indicating difficulties) was not statistically different between sexes (p = 0.082).

While no significant gender differences were observed for emotional problems, peer problems, internalizing scores, or impact scores, the overall trend suggests that males experience more behavioral difficulties, while females demonstrate stronger prosocial behaviors.

### Age-based differences

No statistically significant differences were observed between the two age groups, 3–10 years and 11–14 years, across any domains (Appendix 1). The mean total difficulties scores were similar (13.6 ± 6.7 vs. 13.7 ± 5.7, p = 0.944), as were internalizing (6.7 ± 3.4 vs. 6.2 ± 3.3, p = 0.547) and externalizing scores (6.9 ± 3.9 vs. 7.5 ± 4.2, p = 0.549).

Similarly, individual domains showed no significant differences. Although more younger children exceeded the cutoff for emotional problems (41.2% vs. 24.5%, p = 0.107) and more older children for impact scores (28.6% vs. 17.6%, p = 0.252), these differences were not significant.

### Psychological and behavioral profiles by growth hormone replacement status

Emotional problems were significantly higher in the GH group (4.3 ± 1.8 vs. 3.2 ± 2.3, p = 0.029), while no significant differences were found for total difficulties, internalizing, or externalizing scores. Both groups had similar proportions of children scoring above the cutoff for emotional (31.8% vs. 31.1%, p = 0.954) and peer problems (31.8% vs. 31.1%, p = 0.954). The GH group had a higher proportion scoring below the cutoff for prosocial behavior (27.3% vs. 9.8%, p = 0.073), but this was not statistically significant (**Table 4**).

**Table 4:** Scores of the different domains of the strengths and difficulties questionnaire in children with short stature, by status of growth hormone (GH) replacement.

Domain	Received GH, scores	Did not receive GH, scores	P Value	% Above cut-off^1^		P value
	Mean ± SD	Mean ± SD		Received GH N=22	Did not receive GH N=61	
Emotional problems	4.3 ± 1.8	3.2 ± 2.3	**0.029**	31.8	31.1	0.954
Conduct problems	2.7 ± 1.6	2.9 ± 2.3	0.627	22.7	39.3	0.161
Hyperactivity	5.1 ± 2.2	4.2 ± 2.6	0.172	22.7	16.4	0.528
Peer problems	3.0 ± 1.9	2.9 ± 1.9	0.940	31.8	31.1	0.954
Prosocial	8.0 ± 2.4	8.6 ± 2.0	0.275	27.3	9.8	0.073
Total difficulties	15.0 ± 5.2	13.2 ± 6.4	0.252	36.4	27.9	0.457
Internalizing	7.2 ± 2.9	6.1 ± 3.4	0.181	22.7	23.0	0.983
Externalizing	7.7 ± 3.1	7.1 ± 4.4	0.538	13.6	18.0	0.637
Impact	1.6 ± 2.9	1.4 ± 3.0	0.838	27.3	23.0	0.684

^1^For the Prosocial domain, ‘low’ and ‘very low’ categories were defined as those below the cutoff score of 6.

Values in bold indicate a significant result.

## Discussion

This study offers new insights into the psychological and behavioral challenges faced by children with short stature in Jordan. Our findings indicate a higher prevalence of psychological difficulties among affected children, particularly in conduct, emotional well-being, and peer relationships. More than one-third of the children exhibited clinically significant conduct problems, a rate higher than that reported in the general pediatric population (19). Additionally, approximately one-third of participants experienced emotional difficulties and peer relationship issues, emphasizing the broader psychosocial burden associated with short stature. These findings are consistent with previous research suggesting that short stature may contribute to social exclusion, bullying, and a negative self-image (20). Given the importance of social integration in child development and mental health, these challenges warrant targeted interventions (21).

Our results also highlight notable gender differences in psychological and behavioral outcomes. Males exhibited significantly higher levels of conduct problems, hyperactivity, total difficulties, and externalizing behaviors, with over half exceeding the clinical threshold for conduct disorders and approximately one-third displaying hyperactivity. In contrast, females demonstrated stronger prosocial behaviors, possibly reflecting distinct coping strategies and socialization patterns. These findings align with existing literature indicating that boys are more prone to externalizing behaviors such as aggression and hyperactivity, while girls typically exhibit more prosocial tendencies (22). This discrepancy may be influenced by societal expectations, where greater emphasis on height in males may contribute to increased behavioral difficulties (23). Additionally, boys have been shown to face higher risks of bullying and victimization, further exacerbating negative psychosocial outcomes (24). Biological and hormonal mechanisms may contribute to this disparity. Specifically, higher circulating testosterone levels in boys during early and middle childhood have been associated with a greater tendency toward externalizing behaviors such as aggression and hyperactivity (25). These gender-specific trends underscore the need for tailored interventions, with boys potentially benefiting from behavioral interventions addressing conduct-related issues and hyperactivity, while girls may require support in reinforcing prosocial skills and resilience.

Contrary to the widely held assumption that GH therapy significantly enhances psychosocial well-being (26–28), our study found no significant differences in total difficulties, internalizing, or externalizing scores between children who received GH therapy and those who did not. This finding supports prior evidence suggesting that while GH therapy promotes height gain, its psychological benefits remain inconclusive (4). Of note, children undergoing GH treatment in our study exhibited higher emotional problems, which may reflect the added stress of medical interventions, high treatment expectations, and prolonged clinical monitoring (27); however, causality cannot be inferred from a cross-sectional design. A study presented at the ICE/ENDO 2014 conference found that short but otherwise healthy children receiving GH therapy demonstrated increased depressive symptoms and social withdrawal compared to their untreated peers (29). These findings highlight the importance of a multidisciplinary approach, ensuring that psychological support is integrated into the clinical management of children with short stature to mitigate potential emotional distress.

Given these challenges, a comprehensive, multidisciplinary approach is essential in managing short stature. Beyond endocrinological evaluations and medical interventions, psychological support and parental counseling should be integral components of care (30). Educating parents on the psychosocial challenges their children may face, along with strategies to foster resilience and independence, can help mitigate emotional distress and behavioral difficulties (31). Additionally, public health interventions aimed at reducing societal stigma and bullying related to short stature could enhance the psychological well-being of affected children. Studies have shown that interventions targeting stigma-based bullying can effectively reduce victimization and associated mental health concerns (32). Implementing such strategies could foster a more supportive environment, promoting positive psychosocial outcomes for children with short stature.

This study has several strengths. To our knowledge, this is the first study to investigate the psychological impact of short stature in Jordan, addressing a significant knowledge gap in the region. The use of the SDQ, a standardized and validated tool, ensures comparability with international research. Additionally, integrating both parental reports and medical record data strengthens the validity of findings, offering a more comprehensive assessment of the child’s psychosocial and clinical profile. Furthermore, this study provides valuable insights into gender differences in the psychosocial impact of short stature, emphasizing the need for gender-specific interventions.

However, several limitations should be noted. First, the cross-sectional design prevents causal inference, making it difficult to assess the long-term psychosocial effects of GH therapy or achieved height. Psychosocial adaptation to chronic conditions is dynamic rather than static, necessitating longitudinal studies to evaluate how treatment influences psychological outcomes over time. Second, the sample size was relatively small, which may limit the generalizability of the findings. Third, the study relied on parental reports of psychological difficulties, introducing potential bias. Research suggests that parents often overestimate psychological problems compared to self-reports by children (4). Additionally, the study sample was drawn from a pediatric endocrinology clinic, where parents actively sought medical evaluation for their child’s short stature, potentially leading to an overrepresentation of parents with heightened concerns about psychosocial difficulties. Moreover, GH treatment was not further stratified according to duration, dose or adherence. Finally, the study lacks a control group of children without short stature, which limits the ability to determine whether the observed behavioral difficulties are specifically attributable to short stature. Potential confounding variables may have influenced the observed associations. These include socioeconomic status, which may impact both growth outcomes and psychosocial health; parental mental health, which could shape both the child’s well-being and parent reporting; comorbid chronic illness, which may independently affect psychological outcomes; and exposure to bullying or school difficulties, which may not be directly related to height. Due to the scope of the study and limited sample size, we were unable to adjust for these in multivariable models. We suggest the inclusion of a comparison group in future research.

Another limitation is that, although the Arabic version of the SDQ has been widely used in the Middle East, it has not been formally validated in Jordan. This may limit the precision of the instrument’s cut-offs or subscale interpretations in this specific context. Nevertheless, we used standard scoring methods and thresholds to allow comparison with international studies.

## Conclusion

This study highlights the significant psychosocial burden associated with short stature in children in Jordan, with notable gender differences in behavioral outcomes. While GH therapy remains a widely used intervention, its psychological benefits remain unclear, and children undergoing treatment may experience additional emotional distress. The findings emphasize the need for a multidisciplinary approach that includes psychological support, parental counseling, and public health initiatives aimed at reducing stigma and bullying. Future longitudinal studies are essential to better understand the long-term psychosocial impact of short stature and its management, ultimately guiding more effective interventions.

## Data Availability

The raw data supporting the conclusions of this article will be made available by the authors, without undue reservation.
