# Supplementary material for: Association between short stature and behavioral and emotional difficulties among children in Jordan: a cross-sectional study
Source: Front Endocrinol (Lausanne). 2025 Aug 25;16:1630919. doi: 10.3389/fendo.2025.1630919 (PMC12414789; doi:10.3389/fendo.2025.1630919)
Supplement: Supplementary file 1 [file Table1.docx]

# Supplementary Material

**Table 1 Scores of the different domains of the strengths and difficulties questionnaire in children with short stature, by age group, n 3-10= 34 and n 11-14 =49.**

| **Domain** | **3-10 year olds scores** | **11-14 year olds scores** | **P**  **Value^1^** | **% Above cut-off^1^** | | **P value^2^** |
| --- | --- | --- | --- | --- | --- | --- |
|  | **Mean ± SD** | **Mean ± SD** |  | **3-10 year olds**  **N=34** | **11-14 year olds**  **N=49** |  |
| **Emotional problems** | 3.7 ± 2.4 | 3.3 ± 2.1 | 0.391 | 41.2 | 24.5 | 0.107 |
| **Conduct problems** | 2.7 ± 2.1 | 3.0 ± 2.1 | 0.489 | 29.4 | 38.8 | 0.379 |
| **Hyperactivity** | 4.3 ± 2.4 | 4.5 ± 2.5 | 0.700 | 11.8 | 22.4 | 0.213 |
| **Peer problems** | 2.9 ± 2.0 | 2.9 ± 1.9 | 0.958 | 35.3 | 28.6 | 0.516 |
| **Prosocial** | 8.3 ± 2.5 | 8.5 ± 1.8 | 0.574 | 14.7 | 14.3 | 1.00 |
| **Total difficulties** | 13.6 ± 6.7 | 13.7 ± 5.7 | 0.944 | 26.5 | 32.7 | 0.546 |
| **Internalizing** | 6.7 ± 3.4 | 6.2 ± 3.3 | 0.547 | 26.5 | 20.4 | 0.518 |
| **Externalizing** | 6.9 ± 3.9 | 7.5 ± 4.2 | 0.549 | 14.7 | 18.4 | 0.661 |
| **Impact** | 1.1 ± 2.6 | 1.7 ± 3.2 | 0.340 | 17.6 | 28.6 | 0.252 |

^1^ For the Prosocial domain, ‘low’ and ‘very low’ categories were defined as those below the cutoff score of 6. ^2^ The scores on all domains were tested for age group differences using independent t-test. Age group differences in the percentage of those who scored above the cut-off point were assessed using Chi-square tests. A significance level of .05 was applied to all statistical tests.
